# Bifurcations and Hyperchaos in Mathematical Models of Sleep

**DOI:** 10.3390/bioengineering13070833

**Published:** 2026-07-21

**Authors:** Adriano Scibilia, Luigi Fortuna

**Affiliations:** 1 Institute of Intelligent Industrial Systems and Technologies for Advanced Manufacturing, National Research Council, 20133 Milan, Italy; adriano.scibilia@cnr.it; 2 Department of Engineering, University of Palermo, 90128 Palermo, Italy; 3 Faculty of Electronics Technology, Industrial University of Ho Chi Minh City, Ho Chi Minh City 727000, Vietnam

**Keywords:** sleep dynamics, hyperchaos, nonlinear systems, circadian rhythms, homeostatic regulation, bifurcation theory, intermittency

## Abstract

Sleep regulation is generated by interacting nonlinear feedback loops involving homeostatic pressure, circadian forcing, mutual inhibition, and neuromodulatory mechanisms. This work presents a nonlinear-dynamical interpretation of sleep with emphasis on bifurcation structure, Lyapunov stability, deterministic intermittency, and hyperchaotic fragmentation. After summarizing representative sleep models and the literature on sleep-stage dynamics, we combine bifurcation and Lyapunov exponent analyses of representative sleep-population models with a four-dimensional model in which cortical activation, sleep-promoting activity, homeostatic sleep drive, and the circadian pacemaker form a slow–fast feedback system. The model maps coordinate-dependent feedback, separated homeostatic accumulation and clearance, external sleep-debt forcing, refractory dynamics, and on–off intermittency onto physiological sleep–wake processes. The baseline diagrams identify routes from regular oscillations to period-doubling, chaotic bands, periodic windows, and positive Lyapunov regions, while the new Lyapunov maps of the four-dimensional model identify parameter regions where the largest and second-largest Lyapunov exponents are positive, supporting the hyperchaotic classification. The formulation also shows how chronic sleep debt and disease-associated or stress-related insomnia can be represented as shifts in homeostatic drive, inhibitory gain, and circadian coupling. Bifurcation theory therefore provides a useful framework for interpreting irregular sleep transitions and for inspiring future sleep-technology and digital twin applications.

## 1. Introduction

Sleep is a conserved biological state expressed across a wide range of animal species, but its outward form varies markedly with ecological niche, nervous-system organization, and survival constraints [1,2]. Mammals, birds, insects, and other organisms exhibit sleep-like states with different architectures: some species rely on unihemispheric sleep to preserve vigilance, others show brief REM episodes or short total sleep time, and protected species may sleep for much longer intervals [3,4,5]. This diversity indicates that sleep is not a uniform behavioral template, but an adaptive regulatory process.

Despite this diversity, sleep is commonly identified by a small set of functional properties: behavioral quiescence, increased arousal threshold, reversibility, and homeostatic rebound after deprivation [6]. These features are consistent with major physiological theories of sleep function, including restoration, synaptic homeostasis, and energy conservation [7,8,9]. For the purposes of mathematical modeling, these biological observations imply that sleep should be treated as an active dynamical process rather than as a passive absence of wakefulness.

Dynamical models provide a natural language for studying complex phenomena such as sleep and other human-centered physiological interactions, and a substantial body of literature has developed on this topic [10,11]. Sleep regulation involves nonlinear feedback among homeostatic pressure, circadian forcing, wake-promoting populations, sleep-promoting populations, REM/NREM alternation, and neuromodulatory effects. Such feedback can generate multistability, limit cycles, bifurcations, quasi-periodicity, intermittency, and deterministic chaos. Understanding these mechanisms may help explain irregular sleep patterns, fragile transitions, and pathological sleep fragmentation.

External stressors can bend these physiological trajectories away from healthy consolidated rhythms. Chronic behavioral sleep debt shifts the homeostatic baseline, while disease-associated or stress-related insomnia can produce persistent sleep disruption, hyperarousal, and fragile sleep continuity [12,13]. Post-COVID insomnia provides one recent clinical example of such an external perturbation, but it is used here only as an illustrative stressor rather than as the focal application [14]. At the same time, large-scale digital-health and sleep datasets show how heterogeneous clinical and physiological time series can be organized for prediction, monitoring, and model calibration [15,16]. These observations motivate sleep models that can adapt their parameters to external stressors rather than describing only an isolated healthy oscillator.

This manuscript extends our earlier brief review of sleep dynamical models [17]. The foundational background and two baseline population models recalled in Section 2 and Section 3 are summarized from that work for self-containment. The new contribution of the present paper is concentrated in four elements: a compact four-dimensional hyperchaotic formulation; an explicit separation between homeostatic accumulation and clearance; Lyapunov maps for the four-dimensional parameter plane; and a physiological interpretation of how sleep debt, reduced inhibitory gain, and circadian perturbation can move the system toward fragmented bursting. The aim is therefore not to reproduce the prior survey, but to use it as a compact platform for studying deterministic sleep fragmentation through physiologically interpretable nonlinear mechanisms.

## 2. State of the Art on Sleep Dynamics

The literature on sleep dynamics can be organized around the mechanisms that later appear in the mathematical formulation: homeostatic accumulation, circadian modulation, mutually inhibitory state switching, and nonlinear transitions. Borbély’s two-process model established the interaction between homeostatic sleep pressure and circadian timing as a central organizing principle [18]. Physiologically based sleep–wake models, such as the Phillips–Robinson framework, then connected these drives to mutually inhibitory neural populations that can produce stable wake and sleep states [19]. Related theoretical perspectives, including self-organized criticality, emphasized that sleep-stage transitions may have complex temporal structure rather than purely regular periodicity [20].

Clinical and data-driven studies reinforce the importance of transition structure. Narcolepsy, sleep-onset dynamics, individual differences in sleep architecture, and sleep deprivation all show that the timing and sequencing of state changes carry information beyond total sleep duration [12,21,22,23]. Foundational and quantitative sleep–wake models provide the structural vocabulary for these descriptions, from physiologically based switching circuits to broader mathematical perspectives on two-process regulation [24,25,26]. Recent large-scale and mechanistic studies further connect sleep dynamics to cognitive recovery, brain health, synaptic regulation, REM propensity, and digital phenotyping [13,15,16,27,28,29,30,31]. These works motivate models that can represent both consolidated sleep and fragmented or fragile transitions.

A second strand of literature concerns nonlinear signatures in brain activity and sleep-stage dynamics. Early EEG studies reported low-dimensional chaotic signatures, phase-dependent evoked responses, and positive Lyapunov exponents during sleep or sleep-related brain activity [32,33,34,35,36]. Subsequent thalamocortical, neural-field, and brain-state models showed that nonlinear feedback can generate oscillations, bifurcations, and chaotic transitions near sleep–wake boundaries [37,38,39,40,41,42,43]. Homeostatic and neuromodulatory regulation, including hypocretin/orexin mechanisms, provides an additional physiological route through which nonlinear sleep–wake transitions can be shaped [44]. Complexity- and information-theoretic characterizations of sleep stages, together with recent threshold-based bifurcation models, further support treating sleep regulation as a nonlinear multi-timescale process [45,46]. Slow waves, spindles, circadian entrainment, and thermodynamic or wave-like descriptions of REM–NREM cycling add further evidence that sleep regulation is naturally studied as a nonlinear multi-timescale system [47,48,49,50,51,52].

The present paper builds on this literature by emphasizing two control mechanisms that are especially relevant to fragmentation: inhibitory suppression of cortical activation and homeostatic pressure processing. These mechanisms provide the physiological bridge between established sleep–wake models and the compact hyperchaotic formulation developed below.

## 3. Mathematical Models of Sleep

Two baseline sleep-population models are used to generate the bifurcation and Lyapunov diagrams shown in the next section. Wake activity (*W*), NREM activity (*N*), and REM activity (*R*) are treated as coarse-grained neural populations coupled through nonlinear activation functions. The variables are macroscopic regulatory coordinates rather than direct biochemical measurements. The sigmoid used in both models is

(1) $$\sigma \left(x\right)=\frac{1}{1+\exp[-K_{sig}\left(x-\theta \right)]}.$$

Model 1 is the three-state wake–NREM–REM system

(2) $$\begin{array}{cc} \dot{W} & =-a_{W}W+\sigma \left(I_{W}-b_{WN}N-b_{WR}R+k_{C}C\left(t\right)\right)+\eta_{W}\left(t\right),\\ \dot{N} & =-a_{N}N+\sigma \left(I_{N}-b_{NW}W-b_{NR}R-k_{CN}C\left(t\right)\right)+\eta_{N}\left(t\right),\\ \dot{R} & =-a_{R}R+\sigma \left(I_{R}-b_{RW}W-b_{RN}N+c_{R}N-k_{CR}C\left(t\right)\right)+\eta_{R}\left(t\right), \end{array}$$

where C(t)=sin(2πt/24) is a prescribed circadian modulation and $\eta_{W},\eta_{N},\eta_{R}$ denote optional small numerical perturbations. In the reference simulation from which the Model 1 bifurcation diagram was generated, the nominal values were $a_{W}=0.8$, $a_{N}=0.5$, $a_{R}=0.2$, $b_{WN}=b_{NW}=1.0$, $b_{WR}=b_{RW}=0.5$, $b_{NR}=b_{RN}=0.8$, $c_{R}=0.6$, $k_{C}=0.3$, $k_{CN}=k_{CR}=0.2$, $I_{W}=0.5$, $I_{N}=0.3$, $I_{R}=0.2$, θ=0.5, integration step dt=0.01, time interval 0≤t≤400, and initial condition $\left(W_{0},N_{0},R_{0}\right)=\left(0.11,0.12,0.13\right)$. The bifurcation analysis varies $K_{sig}$ around this nominal setting.

Model 2 extends Model 1 by adding a homeostatic sleep-pressure coordinate *S* and a two-state circadian oscillator $\left(C,\dot{C}\right)$:

(3) $$\begin{array}{cc} \dot{W} & =-a_{W}W+\sigma \left(-b_{WN}N-b_{WR}R+k_{C}C\right),\\ \dot{N} & =-a_{N}N+\sigma \left(\alpha_{S}S-b_{NW}W-b_{NR}R\right),\\ \dot{R} & =-a_{R}R+\sigma \left(c_{R}N-b_{RW}W-b_{RN}N\right),\\ \dot{S} & =\gamma_{W}\left(1-S\right)W-\gamma_{N}SN,\\ \dot{C} & =V,\\ \dot{V} & =\mu_{C}\left(1-C^{2}\right)V-\omega_{C}^{2}C. \end{array}$$

For the Model 2 computations, the nominal parameters were $a_{W}=0.8$, $a_{N}=0.5$, $a_{R}=0.2$, $b_{WN}=b_{NW}=1.2$, $b_{WR}=b_{RW}=0.5$, $b_{NR}=b_{RN}=0.8$, $c_{R}=0.6$, $K_{sig}=14$, θ=0.5, $\alpha_{S}=1.0$, $\gamma_{W}=0.01$, $\gamma_{N}=0.05$, $k_{C}=0.3$, $\mu_{C}=0.2$, $\omega_{C}=2\pi /24$, integration step dt=0.01, time interval 0≤t≤800, and initial condition $\left(W_{0},N_{0},R_{0},S_{0},C_{0},V_{0}\right)=\left(0.1,0.1,0.1,0.1,0,0.01\right)$. The bifurcation diagram varies $b_{WN}$ as the NREM-to-wake inhibitory coupling in the $\dot{W}$ equation over the displayed scan range, while the other parameters remain at their nominal values unless explicitly stated. The Lyapunov map varies $K_{sig}$ together with the baseline/input drive shown on the figure axis.

A nine-state model with subdivided NREM/REM populations and a neuromodulatory coordinate was also present in [17] and remains useful as physiological context. The analysis below, however, focuses on Models 1–2 for baseline bifurcation/Lyapunov evidence and then introduces the four-dimensional hyperchaotic model.

## 4. Dynamical Analysis of Sleep Models

This section moves from representative sleep-population mechanisms to a compact four-dimensional formulation of sleep–wake regulation. The baseline bifurcation and Lyapunov diagrams first show how deterministic irregularity can arise in reduced sleep-population models. The later hyperchaotic formulation then reorganizes wakefulness, sleep-promoting activity, homeostatic pressure, circadian forcing, and neuromodulatory feedback into a slow–fast system designed to emphasize deterministic transitions, intermittent bursts, and parameter-controlled fragmentation.

### 4.1. Bifurcation and Lyapunov Analysis of Baseline Sleep Models

The baseline analyses here shown summarize how representative sleep-population models can move from regular oscillations toward deterministic irregularity as control parameters are varied. They provide us dynamical evidence that sleep-regulatory feedback can support bifurcation cascades, chaotic bands, periodic windows, and regions with positive maximum Lyapunov exponent.

**Figure 1:**
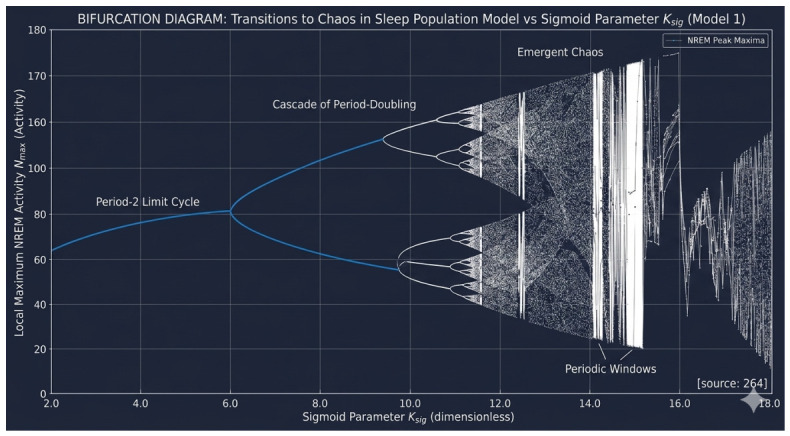
Bifurcation diagram of Model 1 as the sigmoid steepness parameter $K_{sig}$ is varied. The diagram shows a transition from stable periodic oscillations to period-doubling, emergent chaotic bands, and embedded periodic windows.

Figure 1 shows that even the simplest three-variable sleep-population model can lose simple periodicity through a classical route to chaos. Low values of $K_{sig}$ correspond to stable rhythmic behavior, whereas larger values increase neuronal activation sensitivity and produce period-doubling followed by dense chaotic bands. This indicates that deterministic irregularity need not require a high-dimensional sleep architecture; it can already emerge from nonlinear activation gain in a reduced population model.

**Figure 2:**
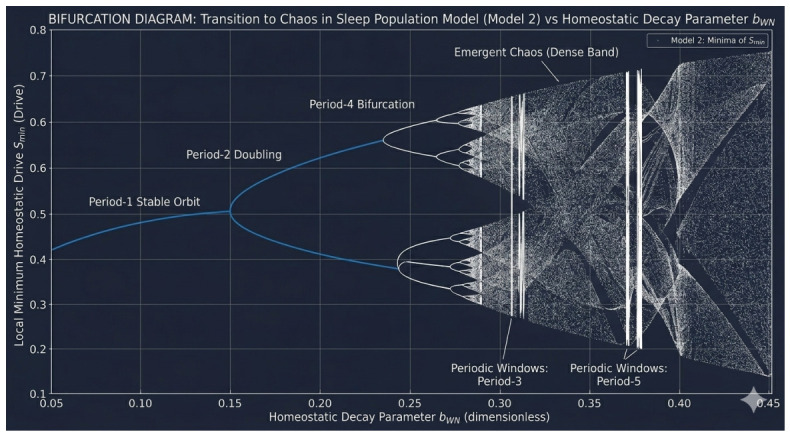
Bifurcation diagram of Model 2 as the NREM-to-wake inhibitory coupling parameter $b_{WN}$ is varied. The diagram displays stable periodic regimes, period-doubling, chaotic bands, and embedded period-3 and period-5 windows.

Figure 2 shows a second route in which NREM-to-wake inhibition, acting within the larger wake–NREM–REM and homeostatic feedback loop, reorganizes the attractor. Increasing $b_{WN}$ moves the model from stable oscillatory behavior toward period-doubled dynamics and then into broad chaotic bands. The visible period-3 and period-5 windows support the interpretation that the irregular regimes arise from deterministic nonlinear structure rather than from numerical noise or purely stochastic forcing.

**Figure 3:**
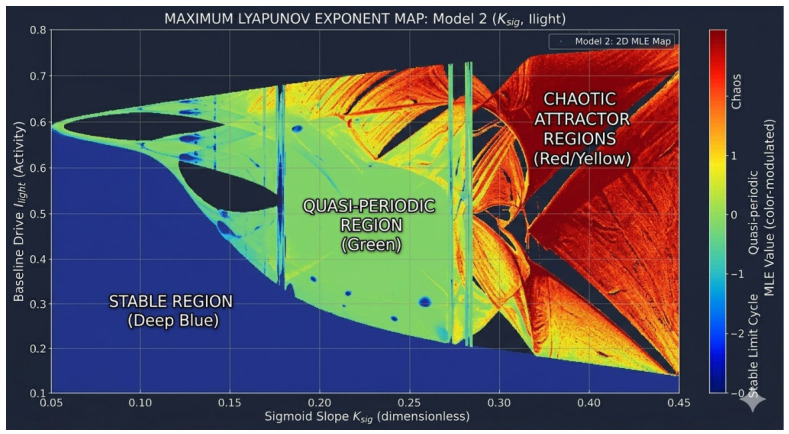
Maximum Lyapunov exponent map for Model 2 across sigmoid steepness $K_{sig}$ and baseline/input drive. Deep blue regions indicate negative maximum Lyapunov exponents and stable attractors; green regions indicate near-zero values compatible with quasi-periodic dynamics; yellow/red regions indicate positive values and chaotic attractors.

Figure 3 provides an independent stability view of the bifurcation results for Model 2. Negative maximum Lyapunov exponents correspond to stable attracting regimes, near-zero values indicate weakly modulated or quasi-periodic behavior, and positive values indicate sensitive dependence on initial conditions. This map supports the baseline claim that established sleep-population models can possess intrinsic deterministic routes to irregular dynamics; it is not used as evidence for hyperchaos in the four-dimensional model introduced below.

Together, Figure 1, Figure 2 and Figure 3 identify two complementary baseline routes to complex sleep dynamics. The first is a gain-driven period-doubling cascade, visible in Model 1 as $K_{sig}$ increases. The second is coupling-driven chaos with embedded periodic windows, visible in Model 2 as $b_{WN}$ varies.

**Figure 4:**
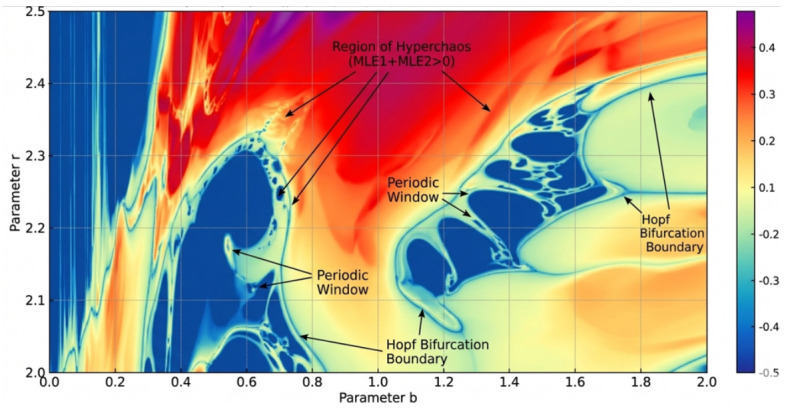
Lyapunov map of the four-dimensional model across inhibitory strength *b* and the second scanned parameter. The color scale reports the maximum Lyapunov exponent. Regions with a positive maximum exponent indicate sensitive dependence on initial conditions; annotated regions where the first and second exponents are jointly positive are classified as hyperchaotic.

**Figure 5:**
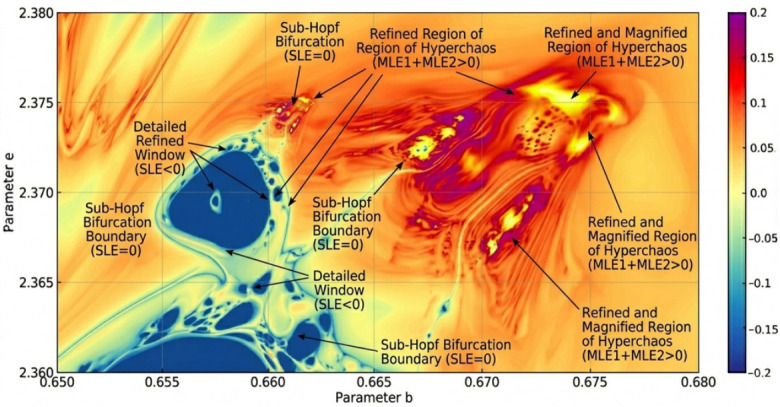
Refined Lyapunov map of the four-dimensional model in the region of strongest bursting. The color scale reports the second-largest Lyapunov exponent. Positive values identify parameter windows in which the second expanding direction is present, supporting the hyperchaotic interpretation of the refined bursting regimes.

These baseline results motivate the compact hyperchaotic formulation introduced next, where the same broad ingredients—activation gain, mutual inhibition, homeostatic processing, and slow circadian modulation—are reorganized into a four-dimensional mechanism for fragmentation. The hyperchaotic evidence for this four-dimensional system is provided by the Lyapunov maps in Figure 4 and Figure 5.

### 4.2. Four-Dimensional Hyperchaotic Sleep Formulation

The four-dimensional formulation maps abstract state variables to physiological sleep–wake components. The variable *W* represents cortical activation or wakefulness; $S_{L}$ represents sleep-promoting neuronal activity, such as VLPO-like sleep-active pathways; *H* represents homeostatic sleep drive or sleep debt; and *C* represents the circadian pacemaker. This mapping frames a hyperchaotic system in sleep-dynamics terms while assigning each coordinate a physiological interpretation. The homeostatic equation separates wake-dependent accumulation from sleep-dependent clearance, and it includes an optional external sleep-debt input used when behavioral or disease-related stressors are modeled.

(4) $$\frac{dW}{dt}=A_{W}-\alpha_{W}W-bS_{L}H+\gamma_{C}C,$$

(5) $$\frac{dS_{L}}{dt}=\rho \left(W-S_{L}\right)-\delta C,$$

(6) $$\frac{dH}{dt}=e_{\mathrm{acc}}W-e_{\mathrm{clr}}S_{L}^{2}+D_{\mathrm{debt}}\left(t\right)-\varphi C-gH,$$

(7) $$\frac{dC}{dt}=\kappa_{H}H-\kappa_{C}C+\kappa_{X}S_{L}C-\mu C^{3},\;\mu >0.$$

For the baseline four-dimensional simulations, $D_{\mathrm{debt}}\left(t\right)=0$. Chronic sleep debt can be represented either by an elevated initial value $H_{0}$ or by a non-negative forcing term $D_{\mathrm{debt}}\left(t\right)$ that raises the homeostatic coordinate over a prescribed interval. Disease-associated sleep disruption can be represented as a parameter perturbation that lowers the inhibitory gain *b*, modifies circadian gains such as $\gamma_{C}$ or $\kappa_{X}$, and shifts the system toward the fragmented-bursting regions shown in the bifurcation diagrams. These mappings are model-based physiological analogues rather than direct clinical diagnoses.

The parameters are summarized in Table 1. Equation (4) describes cortical wakefulness as an intrinsically self-limiting activity with baseline drive $A_{W}$ and decay rate $\alpha_{W}$, suppressed by the combined action of sleep-promoting neurons and accumulated sleep debt, while being modulated by the circadian clock through $\gamma_{C}$. Equation (5) describes activation of the sleep-promoting system as a regulatory response to cortical activation with coupling rate ρ under circadian modulation δ. Equation (6) is the homeostatic pressure equation. With *H* interpreted as sleep debt, the wakefulness term $e_{\mathrm{acc}}W$ accumulates pressure, whereas the quadratic term $-e_{\mathrm{clr}}S_{L}^{2}$ breaks the symmetry of the flow and acts as a one-way clearance valve; the optional term $D_{\mathrm{debt}}\left(t\right)$ represents externally imposed debt. Equation (7) provides the slow circadian governor and introduces the fourth dimension through homeostatic feedback $\kappa_{H}$, circadian self-decay $\kappa_{C}$, bilinear coupling $\kappa_{X}$, and saturating cubic feedback $-\mu C^{3}$ with μ>0.

The model is interpreted on a physiologically admissible numerical domain in which *W*, $S_{L}$, and *H* are non-negative regulatory coordinates and *C* remains a bounded circadian state. The polynomial equations are not intended to describe arbitrary excursions in $R^{4}$; rather, the displayed trajectories are retained only after transient removal and after numerical checks that the orbit remains bounded in the plotted parameter region. Parameter scans that leave this admissible bounded domain are treated as divergent or unstable pathological regimes rather than as physiologically interpretable sleep states.

#### Numerical Implementation and Lyapunov Diagnostics

The four-dimensional system was integrated using a fixed-step fourth-order Runge–Kutta scheme with step size Δt=0.005. Unless otherwise stated, the nominal initial state was $\left(W_{0},S_{L0},H_{0},C_{0}\right)=\left(0.1,0.05,0.6,0.2\right)$ and $D_{\mathrm{debt}}\left(t\right)=0$. Bifurcation scans were obtained by varying one control parameter at a time while keeping the remaining parameters fixed at their nominal values, discarding an initial transient interval before sampling the local extrema of W(t) on the asymptotic trajectory. The displayed diagrams therefore plot steady-state extrema of the cortical activation coordinate rather than raw transient time series.

**Figure 6:**
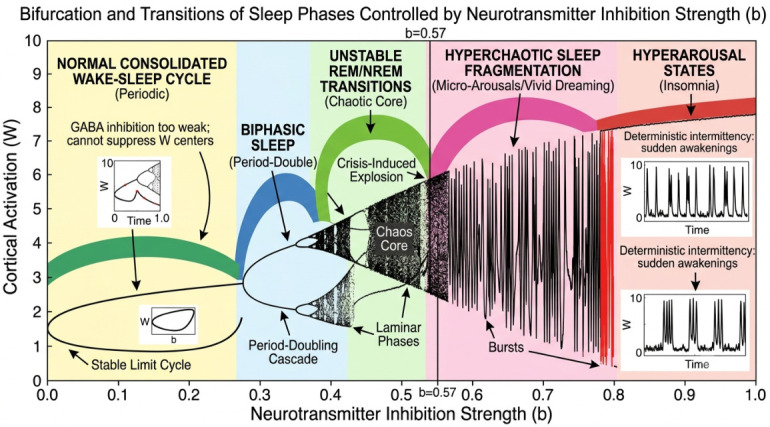
Bifurcation behavior of cortical activation *W* in Equations (4)–(7) as the inhibitory strength parameter *b* is varied. The plotted points represent sampled extrema of W(t) after transient removal. Physiological labels denote model analogues of consolidated sleep, fragmentation, and hyperarousal rather than direct clinical classification.

**Figure 7:**
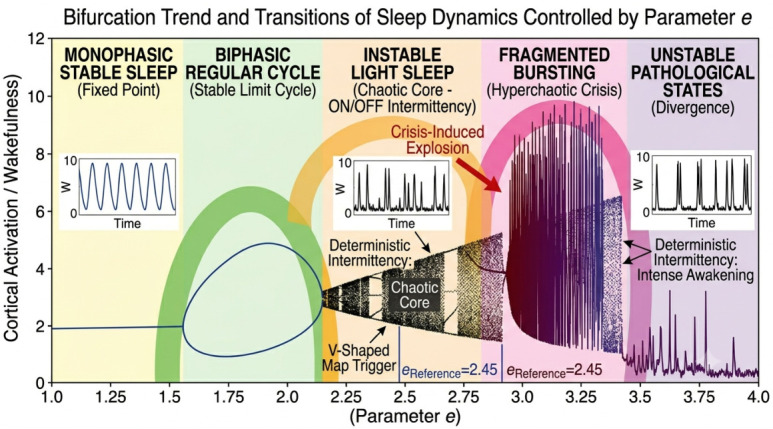
Bifurcation behavior of cortical activation *W* in Equations (4)–(7) as the homeostatic clearance gain $e_{\mathrm{clr}}$ is varied. The plotted points represent sampled extrema of W(t) after transient removal and illustrate stable sleep-like oscillations, biphasic cycling, fragmented bursting near crisis, and unstable regimes.

For the inhibitory-axis scan, *b* was varied over the range displayed in Figure 6; for the homeostatic-axis scan, $e_{\mathrm{clr}}$ was varied over the range displayed in Figure 7 while $e_{\mathrm{acc}}$ was kept fixed. The Lyapunov maps in Figure 4 and Figure 5 were computed on the same four-dimensional equations by evolving the tangent dynamics along the numerical trajectory, orthonormalizing perturbation vectors, and averaging exponential growth rates after transient removal. In this convention, ordinary chaos is indicated by a positive maximum Lyapunov exponent, whereas hyperchaos is identified only where both the largest and second-largest exponents are positive.

This formulation emphasizes five mechanisms relevant to sleep architecture: slow–fast switching between cortical activation and slow regulatory variables; accumulation and rapid release of homeostatic pressure; broken symmetry in the homeostatic coordinate; refractory intervals imposed by the slow controller; and on–off intermittency, in which quiet low-amplitude dynamics are punctuated by sudden high-amplitude bursts. The intended parameter domain uses positive decay and gain parameters, μ>0, and trajectories that remain in a bounded numerical attractor after transient removal. In physiological terms, these bursts are deterministic analogues of micro-arousals or abrupt awakenings, while the laminar phases correspond to consolidated rest intervals.

### 4.3. Bifurcation Structure Controlled by Inhibition and Homeostatic Processing

Figure 6 illustrates the bifurcation structure of the model under variation in the inhibitory parameter *b*. This parameter represents neurotransmitter sensitivity: it measures how effectively sleep-promoting GABAergic pathways suppress cortical activation. At high values of *b*, the model exhibits a consolidated oscillatory regime analogous to stable sleep with strong inhibitory control. As *b* is reduced, the trajectory passes from biphasic cycling into unstable REM/NREM-like transitions through period-doubling and crisis-induced expansion. In the intermediate low-inhibition region, the diagram identifies a hyperchaotic sleep-fragmentation regime supported by the Lyapunov evidence in Figure 4 and Figure 5. In this region, the trajectory remains near a quiet chaotic core before producing sudden awakening-like bursts, a deterministic analogue of micro-arousals or vivid-dreaming interruptions. For very low inhibition, the model approaches a hyperarousal-like state, consistent with weakened sleep-promoting suppression.

Figure 7 illustrates the complementary bifurcation route of the model controlled by the homeostatic clearance gain $e_{\mathrm{clr}}$ while accumulation is governed separately by $e_{\mathrm{acc}}$. Low values of $e_{\mathrm{clr}}$ correspond to a slow, monophasic pressure-clearance profile, while intermediate values correspond to a stable biphasic regular cycle. In the intermediate-to-high processing region, the diagram marks a crisis-induced expansion: pressure processing becomes sufficiently fast that the homeostatic variable behaves like a volatile capacitor, repeatedly forcing cortical activation out of sleep through high-frequency bursts. At larger values, fragmented bursting develops into unstable regimes. Together, Figure 6 and Figure 7 illustrate two interpretable routes to fragmentation in the model: weakening inhibitory suppression and accelerating homeostatic clearance.

## 5. Hyperchaotic Interpretation and Mechanism

The mathematical economy of the hyperchaotic formulation lies in how a small number of nonlinear terms generate biologically recognizable sleep phenomena. The term $S_{L}^{2}$ in Equation (6) breaks the symmetry of the flow and acts as a one-way pressure-clearance valve. Sleep pressure can build silently during wakefulness through the $e_{\mathrm{acc}}W$ contribution or through $D_{\mathrm{debt}}\left(t\right)$, but once sleep-promoting activity exceeds a threshold, the $-e_{\mathrm{clr}}S_{L}^{2}$ contribution lets the system discharge in a preferred direction. This explains why the bifurcation diagrams display sudden directional bursts rather than smooth sinusoidal oscillations.

A second important property is low-amplitude internal activity during apparently quiet sleep. Traditional phenomenological models often treat sleep as a nearly flat inactive state. In the present interpretation, the quiet phase is itself a low-amplitude chaotic core: cortical activation may remain low, but the underlying homeostatic and circadian variables continue to evolve. This is consistent with the biological view of sleep as an active state rather than a passive absence of wakefulness.

The fourth coordinate also provides a refractory mechanism. The circadian pacemaker variable *C* does not merely force a 24 h rhythm; it introduces a slow memory that prevents immediate symmetric return after a burst. In sleep terms, the system must pass through a reset interval before another large transition can occur. This slow recovery explains why the model can produce laminar periods interrupted by sudden spikes, a hallmark of on–off intermittency.

The two principal bifurcation axes have a compact physiological interpretation. Parameter *b* is the inhibitory axis: it controls how strongly sleep-promoting pathways suppress cortical activation. Parameter $e_{\mathrm{clr}}$ is the clearance axis: it controls how rapidly sleep-promoting activity removes accumulated homeostatic pressure, while $e_{\mathrm{acc}}$ and $D_{\mathrm{debt}}\left(t\right)$ set the accumulation load. Their interaction places consolidated sleep, light biphasic cycling, fragmented sleep, and hyperarousal-like regimes in different regions of a bifurcation landscape rather than in unrelated dynamical states.

## 6. Discussion

The analysis shifts the emphasis from visual complexity to dynamical mechanisms. The baseline bifurcation and Lyapunov figures show explicit routes from regular oscillations to chaotic regimes, while the four-dimensional formulation provides a compact mechanism for fragmentation. Sleep regulation contains classical ingredients of bifurcation phenomena: mutual inhibition between wake- and sleep-promoting populations, saturation nonlinearities, slow homeostatic pressure, circadian forcing, and neuromodulatory feedback loops. The four-dimensional hyperchaotic interpretation adds coordinate-dependent feedback, separated accumulation and clearance, external debt forcing, and a slow refractory controller. These mechanisms generate limit cycles, attractor deformation, period-doubling cascades, crisis-induced expansion, on–off intermittency, and hyperchaotic bursting.

From a mathematical standpoint, the baseline bifurcation and Lyapunov diagrams provide structural evidence of deterministic regime changes in representative sleep-population models, while the four-dimensional Lyapunov maps show how the new equations organize bursting through the inhibitory and homeostatic control axes. Figure 4 identifies broad regions with a positive maximum Lyapunov exponent, and Figure 5 refines the same parameter neighborhood by showing where the second-largest exponent is also positive. The hyperchaotic label is therefore restricted to regions in which two expanding Lyapunov directions are present.

From a physiological perspective, bifurcation points represent dynamical analogues of fragile sleep regulation. Near such parameter values, small changes in inhibitory gain, homeostatic pressure processing, circadian input, or mutual inhibition produce large qualitative changes in sleep architecture. Chronic sleep debt can be modeled as a positive shift in $H_{0}$ or as a non-negative $D_{\mathrm{debt}}\left(t\right)$ input; disease-associated or stress-related insomnia can be modeled as reduced inhibitory gain *b* together with altered circadian coupling. These mappings connect external stressors to movement across the bifurcation landscape, while direct clinical validation remains a target for future data-driven studies.

The reduced model should also be distinguished from established physiologically detailed frameworks such as Phillips–Robinson-type sleep–wake systems. Those models track specific neurophysiological drives and mutually inhibitory populations with greater biophysical detail. The present formulation instead trades biochemical resolution for a low-dimensional topological view of how state transitions, fragmentation, and refractory recovery can arise from deterministic geometry. This makes the model complementary to, rather than a replacement for, detailed sleep-stage models.

Validating these nonlinear configurations requires a bridge between differential equations and empirical clinical time series. Digital-health analytics and data-driven sleep-modeling studies provide a relevant blueprint for extracting multiscale features from heterogeneous data streams [15,16]. Large-scale polysomnography and wearable actigraphy datasets, including clinical insomnia or sleep-fragmentation cohorts, could be used to map EEG power bands, heart-rate variability, and movement bursts onto the abstract coordinates $\left(W,S_{L},H\right)$ and to estimate parameters such as *b*, $e_{\mathrm{acc}}$, and $e_{\mathrm{clr}}$ as latent patient-specific quantities. Multimodal physiological-state inference methods provide an additional route for connecting EEG, autonomic, and behavioral data to the model variables [53]. In this setting, the four-dimensional system becomes a modeling engine for patient-specific digital twins of sleep fragmentation.

For sleep technology, the results indicate that nonlinear-dynamics tools can support sleep prediction, adaptive stimulation, and digital twin models of sleep regulation. If a subject-specific model approaches a bifurcation boundary, stimulation or behavioral interventions can be designed to stabilize desired oscillatory regimes. Conversely, chaos-control methods provide inspiration for regulating pathological transitions in engineered or neurophysiological systems.

## 7. Conclusions

This work provides a nonlinear-dynamics perspective on mathematical models of sleep, with particular emphasis on bifurcations, Lyapunov stability, and hyperchaotic sleep fragmentation. The baseline diagrams show routes from stable oscillations to period-doubling, chaotic bands, embedded periodic windows, and positive Lyapunov regions. The four-dimensional hyperchaotic formulation then maps cortical activation, sleep-promoting activity, homeostatic pressure, and circadian control onto a compact system capable of generating deterministic intermittency and burst-like awakenings.

The revised formulation separates wake-driven homeostatic accumulation from sleep-promoting clearance, adds an optional sleep-debt forcing term, and identifies how reduced inhibitory gain and perturbed circadian coupling can move the system toward fragmentation. The new Lyapunov maps support the hyperchaotic interpretation in parameter regions where both the largest and second-largest Lyapunov exponents are positive. Reducing inhibitory strength *b* moves the model from consolidated sleep toward biphasic cycling, chaotic cores, hyperchaotic bursting, and hyperarousal-like regimes, whereas increasing the clearance gain $e_{\mathrm{clr}}$ provides a complementary route in which rapid pressure processing destabilizes quiet sleep and produces repeated awakening-like bursts.

These results show that sleep fragmentation can emerge from deterministic nonlinear geometry rather than only from external stochastic perturbations. They also show how external factors such as chronic sleep debt and disease-associated insomnia can be represented as interpretable shifts in homeostatic load, inhibitory suppression, and circadian coupling. Future work should estimate bifurcation structures directly from physiological sleep recordings, compare model-derived stability landscapes with EEG-based complexity measures, and investigate whether patient-specific parameter shifts can predict fragile sleep transitions. The results indicate that bifurcation theory provides a unifying framework linking sleep regulation, irregular sleep transitions, digital-health analytics, and the emergence of complex neurophysiological behavior.

## Figures and Tables

**Table 1:** Parameters and initial-condition notation of the hyperchaotic model in Equations (4)–(7).

Symbol	Role
$A_{W}$	baseline cortical drive
$\alpha_{W}$	cortical decay rate
*b*	inhibitory strength associated with the $S_{L}H$ suppression term; bifurcation axis for inhibitory control
$\gamma_{C}$	circadian modulation gain on cortical activation *W*
ρ	coupling rate between cortical activation and sleep-promoting activity
δ	circadian modulation of sleep-promoting activity $S_{L}$
$e_{\mathrm{acc}}$	wake-dependent homeostatic accumulation gain
$e_{\mathrm{clr}}$	sleep-promoting homeostatic clearance gain and bifurcation axis for pressure processing
$D_{\mathrm{debt}}\left(t\right)$	optional external sleep-debt forcing; set to zero in baseline simulations
φ	circadian coupling into the homeostatic coordinate *H*
*g*	homeostatic decay rate
$\kappa_{H}$	homeostatic feedback into the circadian coordinate *C*
$\kappa_{C}$	circadian self-decay
$\kappa_{X}$	bilinear coupling between sleep-promoting activity and the circadian coordinate
μ	positive cubic circadian saturation coefficient in the term $-\mu C^{3}$
$\left(W_{0},S_{L0},H_{0},C_{0}\right)$	initial state of the four-dimensional trajectory
integrator/step/horizon	numerical scheme used for time-domain and bifurcation calculations

## Data Availability

No new data were created or analyzed in this study. Data sharing is not applicable to this article.

## Abbreviations

The following abbreviations are used in this manuscript:

EEG	Electroencephalography
NREM	Non-rapid eye movement
REM	Rapid eye movement
